# The Limits of Effective Degrees of Freedom in UCA based Orbital Angular Momentum Multiplexed Communications

**DOI:** 10.1038/s41598-020-61329-z

**Published:** 2020-03-23

**Authors:** Zhipeng Li, Fengzhong Qu, Yan Wei, Guowei Yang, Wen Xu, Jing Xu

**Affiliations:** 1 Ocean College, Zhejiang University, Zhoushan, Zhejiang 316021 China; 2 School of Communication Engineering, Hangzhou Dianzi University, Hangzhou, Zhejiang 310000 China

**Keywords:** Electrical and electronic engineering, Acoustics

## Abstract

Orbital angular momentum (OAM) multiplexing technique has recently emerged and generated widespread interests since the OAM was discovered as a property of electromagnetic wave and acoustic wave. It was widely acknowledged that OAM multiplexing can achieve very high effective degrees of freedom (EDOF) and improve the spectral efficiency in optical, radio and acoustic communications. However, in the field of free-space optical (FSO) communications, it was demonstrated that OAM multiplexing is not the optimal multiplexing technique and the spatial bandwidth product (SBP) limits the EDOF. Is there any EDOF limits of OAM multiplexing in radio and acoustic communications? Could OAM multiplexing be safely scaled to far field? Here, we discover that the azimuthal resolution of OAM mode generator in OAM multiplexing limits its EDOF. Furthermore, we also verify that the OAM multiplexing in radio and acoustic communication fails to enable a long distance transmission and high EDOF simultaneously incurred by the inherently imperfect OAM mode generator.

## Introduction

It was recently discovered that orbital angular momentum (OAM) and spin angular momentum (SAM) jointly compose the angular momentum of photons, where the OAM is a brand new fundamental angular momentum carried by twisted photons^1–3^. While the SAM only has two orthogonal states which correspond to the two states of polarization^4^, the OAM has an unlimited number of orthogonal states due to its helical phase term $\exp (-il\phi )$, where *l* indicates the topological charges and *ϕ* is the azimuthal angle^1^. The realization of waves carrying OAM has led tentative applications which range from micro-scale manipulation and quantum, to communications. In the field of micro-scale manipulation, the interaction between the OAM beam and matter is applied in particle manipulation, such as optical tweezers and acoustic tweezers^5–7^, and the acoustic vortex carrying OAM is capable of rotating a millimetre-size suspended disk at a steady frequency, which is caused by the angular momentum induced radiate torque^8,9^. In the field of quantum, the entanglement of OAM states has potential in quantum cryptography and quantum communications^10,11^.

The promising application of OAM to communications has drawn extensive studies on OAM^3,12^. Note that waves carrying OAM in communications could enable certain advantages, such as (a) improving spectral efficiency^13–16^; (b) increasing channel capacity^15–17^; (c) achieving secure communication^18^; (d) realizing optical switch^19,20^. Recent reports have verified the application of OAM multiplexing in communications. It was demonstrated that the coaxially transmitted optical OAM beams sharing the same frequency, phase lag, and polarization are capable of transmitting multiple independent data streams in free-space optical (FSO) communications^17^. Based on this result, combining the OAM multiplexing with classical multiplexing was verified to yield a further increase of the channel capacity. For instance, in the FSO communications, a terabit transmission was experimentally realized with the combination of the OAM multiplexing and polarization multiplexing^16^. Subsequently, Huang et al. accomplished the concept demonstration of 100*Tbits*∕*s* FSO communications by jointly using the OAM multiplexing, wavelength division multiplexing (WDM) and polarization multiplexing^15,21,22^. More than the thriving application in FSO communications, the OAM multiplexing has witnessed the exciting employments in millimetre wave (mm-wave) communications and acoustic communications, since it was demonstrated to enhance the spectral efficiency and the channel capacity^13,14,23^. For instance, a near field OAM multiplexed mm-wave communications was verified to realize a 32*Gbits*∕*s* rate data transmission^14^. In the field of acoustic communication, a uniform circular array (UCA) based near field OAM multiplexed acoustic communications increases the spectral efficiency up to 8.0 ± 0.4(bit/s)/Hz^13^.

Nevertheless, many factors do exist and affect the performance of OAM multiplexed communications. Like in free space, the OAM beams are distorted by the turbulent medium^22,24,25^. Even without the turbulent medium-induced disturbance, the OAM multiplexing is still not a flawless technique. For example, the physical resource, described by spatial bandwidth product (SBP) in FSO communications, limits the effective degrees of freedom (EDOF) and the detectable power of optical signals^26^. Is there any factor limiting the OAM multiplexing in radio and acoustic communications as well? In addition, although the near field applications of radio and acoustic OAM multiplexed communications have been successfully verified and some believed that it could be safely scaled to long distance in the far-field^13,14,23^, we consider it is worthwhile to verify the far-field applications.

In this article, to tackle the first question, the physical resource of the uniform circular array (UCA) is investigated (here we use UCA as OAM mode generators and detectors) by verifying the mutual orthogonality between OAM modes theoretically and numerically, where the normalized spiral spectrum is utilized to portray the composition of OAM modes. And we discover that the azimuthal resolution of OAM mode generators and detectors limits the EDOF in OAM multiplexing. Further more, we find that the EDOFs would gradually decrease down to 3 as the transmission distance increases. Given the inevitable flaw of UCA, the decrease of EDOFs and the inherent severe power attenuation, we conclude that OAM multiplexing in radio and acoustic communications is incapable of realizing high EDOF and long transmission distance simultaneously. This work is expected to be beneficial to the design and implementation of practical UCAs based OAM multiplexed communications.

## EDOF limits-physical resource of UCA-based OAM mode generator and detector

In the radio domain and the acoustic domain, spiral phase plate (SPP) and UCA are widely utilized to generate OAM modes^9,27,28^. Using SPP can generate only one OAM mode with a constant frequency in the medium which has a constant refractive index^28,29^, while the UCA was proved to generate many OAM modes at the same time in both radio domain and acoustic domain^8,27^. Given the flexibility and the simple structure of UCA, we use UCA as OAM mode generators and detectors in simulations.

Firstly, we analyze the physical resource of UCAs when they are used as OAM mode generators. As a typical UCA structure, single-ring ${{\rm{UCA}}}_{(1\times {N}_{t})}$ is a single annulus consisting of *N*_*t*_ uniform circular arranged elements. Its azimuthal resolution is equal to the number of elements *N*_*t*_ and the constant azimuth interval between adjacent elements is $\Delta \phi =\frac{2\pi }{{N}_{t}}$. In order to generate the OAM mode *l*, the signal component induced by the *n*th element (anticlockwise direction, locates at *ϕ*_*n*_ on transmitter plane) at distance *z* is $\frac{{A}_{n}\lambda }{4\pi z{{\prime} }_{n}}\exp (-ikz{{\prime} }_{n})\exp (-il{\phi }_{n})$. Both the radio and acoustic OAM mode *l* generated by the UCA_1×*Nt*_ is given by

1 $$u(r,z,\phi ,l)={\sum }_{n=1}^{Nt}\frac{{A}_{n}\lambda }{4\pi z{{\rm{{\prime} }}}_{n}}\exp (i\omega t-ikz{{\rm{{\prime} }}}_{n})\exp (-il{\phi }_{n}),$$

where (*r*, *ϕ*) denotes the polar coordinates of the point on the receiver plane, *z* is the distance between transmitter plane and receiver plane, *l* is topological charge, *N*_*t*_ is the number of elements, $\frac{\lambda }{4\pi {z}_{n}^{{\prime} }}$ denotes the space loss, *A*_*n*_ is the *n*th element induced amplitude, *A*_1_ = *A*_2_ = … = *A*_*n*_ because of the rotational symmetry of OAM modes, $k=\frac{2{\rm{\pi }}}{\lambda }$is wavenumber,$\ z{{\prime} }_{n}=\sqrt{{z}^{2}+{r}^{2}+{r}_{n}^{2}-2{r}_{n}r\cos (\phi -{\phi }_{n})}$  is the distance between *n*th element and the point (*r*, *ϕ*) on the receiver plane, (*r*_*n*_, *ϕ*_*n*_) denotes the polar coordinates of *n*th elements, *r*_1_ = *r*_2_ = … = *r*_*n*_, *r*_*n*_ is a constant, ${\phi }_{n}=\frac{2\pi n}{{N}_{t}}$ is the azimuthal position of *n*th element and *z* denotes the distance between transmitter plane and receiver plane. The mutual orthogonality of the OAM modes generated by UCA_1×*Nt*_ can be verified by calculating the inner product which is given by

2 $$\begin{array}{ccc}u(r,z,m)\cdot {u}^{\ast }(r,z,k) & = & \int r{\rm{d}}r{\rm{d}}\phi u(z,r,\phi ,m)\times {u}^{\ast }(r,z,\phi ,k)\\  & = & {\int }_{0}^{+{\rm{\infty }}}{\int }_{0}^{2\pi }r{\rm{d}}r{\rm{d}}\phi {\sum }_{n=1}^{{N}_{t}}\frac{{A}_{n}\lambda }{4\pi z{{\rm{{\prime} }}}_{n}}\exp (i\omega t-ikz{{\rm{{\prime} }}}_{n})\exp (-im{\phi }_{n})\\  &  & \times {\sum }_{h=1}^{Nt}{\left(\frac{{A}_{h}\lambda }{4\pi z{{\rm{{\prime} }}}_{h}}\right)}^{\ast }{\exp }^{\ast }(i\omega t-ikz{{\rm{{\prime} }}}_{h}){\exp }^{\ast }(-ik{\phi }_{h}),\end{array}$$

If *m* = *κ* × *N*_*t*_ + *k*, then $\exp (-im{\phi }_{n})=\exp (-ik{\phi }_{n})$ and the inner product of *u*(*r*, *z*, *m*) and u*(*r*, *z*, *k*) is non-zero, where *κ* is an integer. Given that the mutual orthogonality is independent of the transmission distance *z*, assume *z* = 0 and calculate the inner product between generated OAM modes at transmitter plane. Simplifying the Eq. (2) yields the following:

3 $$\begin{array}{ccc}u(r,z,m)\cdot {u}^{\ast }(r,z,k) & = & {\sum }_{n=h=1}^{{N}_{t}}\frac{{A}_{n,z=0}{A}_{h,z=0}^{\ast }{\lambda }^{2}}{{(4\pi z)}^{2}}\exp (-im{\phi }_{n}){\exp }^{\ast }(-ik{\phi }_{h})\\  & = & \{\begin{array}{cc}0 & m-k\ne \kappa {N}_{t},\\ {N}_{t}\frac{||{A}_{n,z=0}\lambda |{|}_{2}^{2}}{{(4\pi z)}^{2}} & m-k=\kappa {N}_{t},\end{array}\end{array}$$

where *κ* is an unbounded integer. Eq.  (3) implies the periodicity of the OAM modes generated by ${{\rm{UCA}}}_{(1\times {N}_{t})}$. If *N*_*t*_ → *∞*, this periodicity of Eq.  (3) will vanish, and for a constant *m*, the *k* that subjects to *m* − *k* = 0 is the sole solution to the non-zero inner product. This result is similar to the definition of the inter-modal crosstalk of OAM modes^25^. If *N*_*t*_ is a finite integer, for a constant *m*, any *k* that subjects to *m* − *k* = *κN*_*t*_ and *κ* ∈ *Z* is the solution to the non-zero inner product.

**Figure 1:**
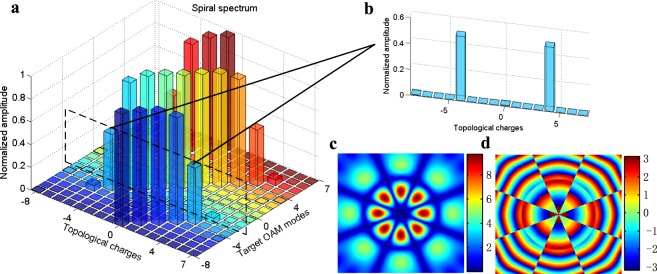
(**a**) Normalized spiral spectrum of OAM modes generated by UCA_(1×8)_; (**b**) spiral spectrum of UCA_(1×8)_ generated mode “*l* = −4”; (**c**) intensity pattern of the mode “*l* = −4”; (**d**) phase pattern of mode “*l* = −4”.

**Figure 2:**
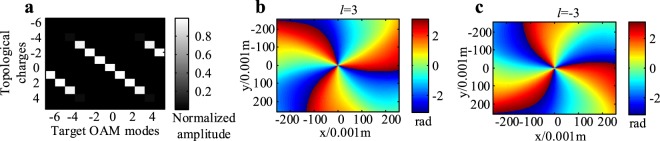
(**a**) Normalized spiral spectrum of OAM modes generated by UCA_1×7_; (**b**) phase pattern of mode *l* = 3; (**c**) phase pattern of mode *l* = −3.

Actually, the maximum number of mutual orthogonal modes generated by a ${{\rm{UCA}}}_{(1\times {N}_{t})}$ is equal to the *N*_*t*_ (see methods). When *N*_*t*_ is odd, this result can be perfectly explained by Nyquist-Shannon sampling theorem, because the maximum number of OAM modes is *N*_*t*_ and the maximal OAM mode is $|l| < \frac{{N}_{t}}{2}$. When *N*_*t*_ is even, at the range boundary, i.e. $|l|=\frac{{N}_{t}}{2}$, further discussion is needed. Note that, when *N*_*t*_ is even, the UCA generated modes $l=\pm \,\frac{{N}_{t}}{2}$ are not pure OAM modes, but are the superposition of pure OAM modes (the theoretical analysis is presented in the section “Methods”). In [1] and [2], this impure OAM modes was not considered as EDOF. However, since the impure OAM mode is orthogonal to other OAM modes, we think this impure OAM mode $l=+\,\frac{{N}_{t}}{2}$ or $l=-\,\frac{{N}_{t}}{2}$ is efficient to carry a independent data stream as other EDOF do. Figure 1 shows an example of *UCA*_1×8_ generated “target OAM modes”. The columns sharing the same color in Fig. 1(a) indicate the spiral spectrum of the corresponding target OAM mode. Figure 1(a) suggests that the *UCA*_1×8_ can only generate 7 pure OAM modes. In Fig. 1(b), the spiral spectrum also suggests that the target OAM modes ${l}_{t}=\pm \,\frac{{N}_{t}}{2}$ (*l*_*t*_ means the topological charge of target OAM mode) are the superposition of pure OAM modes $l=+\,\frac{{N}_{t}}{2}$ and $l=-\,\frac{{N}_{t}}{2}$, respectively. From the aspect of intensity and phase pattern, modes *l*_*t*_ = ±4 are very similar to the superposition of Laguerre-Gaussian (LG) modes with topological charges *l* = −4 and *l* = +4 (see supplementary). When *N*_*t*_ is odd, each OAM mode generated by ${{\rm{UCA}}}_{(1\times {N}_{t})}$ has become pure. Figure 2 shows an example of *N*_*t*_ = 7 and illustrates the normalized spiral spectrum of the OAM modes generated by UCA_(1×7)_ and the phase pattern of OAM modes *l*_*t*_ = ±3. Therefore, for any single-ring UCAs used as OAM mode generators, we obtain that its physical resource is equal to the number *N*_*t*_ which is its corresponding azimuthal resolution, and the EDOF of OAM multiplexing should be less than or equal to the number *N*_*t*_.

**Figure 3:**
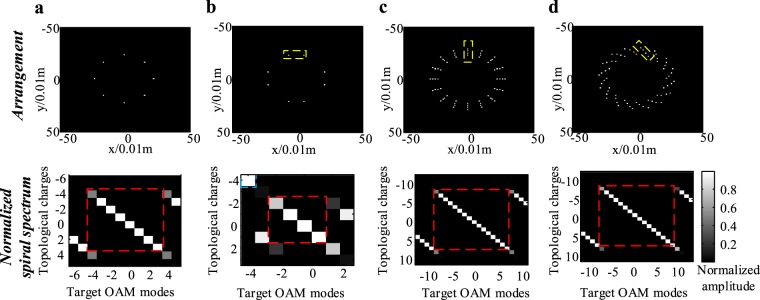
First row shows the arrangements of single-ring UCA_1×8_, unequal interval arranged single-ring UCA_1×8_, four rings UCA_4×16_ and vortex shaped four rings UCA_4×16_ respectively; second row shows the corresponding normalized spiral spectrum of 4 UCAs.

For unequally spaced UCAs and multi-ring UCAs which can also generate OAM modes^13,27^, we can determine their physical resources by converting them into single-ring UCAs. For the unequally spaced UCA with rotational symmetry, such as the UCA_(1×8)_ shown in Fig. 3(b), it can be segmented into four independent and rotational symmetric cells which consist of two elements, as shown in the dashed-line box. Therefore, the unequally spaced UCA_1×8_ can be regarded as a special single-ring UCA_1×4_ with four elements. Furthermore, the UCA_(1×8)_ can also be considered as the superposition of two equally spaced single layer UCA_(1×4)_. Note that both of these two approaches can obtain the infimum of physical resources of unequally spaced UCAs. Since the physical resource of single layer UCAs is equal to the number *N*_*t*_ as we proved earlier, the physical resource of this unequally spaced UCA_(1×8)_ is at least 4. If the intervals between adjacent elements of equally spaced UCAs and that of unequally spaced UCA have a negligible difference, the physical resource of unequally spaced UCA will be greater than the number of cells under a short transmission distance (if under a long transmission distance, this negligible difference will be magnified and the physical resource will be reduced to 4). In Fig. 3(b), as shown in the dashed-line box in the normalized spiral spectrum, the aggregated physical resource of the unequally spaced UCA_(1×8)_ is 5 rather than 4. We can also obtain the physical resources of multi-ring UCAs. Figure 3(c) is an example of multi-ring UCA_4×16_ which can be segmented into sixteen rotational symmetric cells and each cell comprises of four radial distributed elements. This multi-ring UCA_4×16_ can also be regarded as the superposition of four single-ring UCA_(1×16)_ with four different radii. Hence, its physical resource should be 16. The corresponding normalized spiral spectrum shown in Fig. 3(c) verifies the prediction that the physical resource of this multi-ring UCA_4×16_ is equal to 16.

In fact, the physical resources of UCAs can be decomposed into azimuthal resolution and radial resolution, where the former limits the signal variation in azimuthal direction and the later limits the signal variation in radial direction. For instance, for single-ring UCA_1×8_ in Fig. 3(a), and its azimuthal resolution is 8 and its radial resolution is 1, while the azimuthal resolution of multi-ring UCA_4×16_ in Fig. 3(c) is 16 and its radial resolution is 4. Since in OAM multiplexing, the mutual orthogonality of OAM modes derives from the transverse helical phase pattern, the EDOF depends on the azimuthal resolution of UCA rather than its radial resolution. From this, the radial resolution helps little in increasing the EDOF in OAM multiplexing, but could be used in spatial-mode multiplexing which requires the radial intensity variation of signals. Figure 3(d) shows an example of vortex shaped UCA_(4×16)_, and the corresponding normalized spiral spectrum shows that its physical resource is 16. Therefore, for unequally spaced UCA and multi-ring UCA, by converting them into single-ring UCA, we can obtain the physical resource which is equal to the azimuthal resolution of single-ring UCA. Given that, for any UCA used as OAM mode generator, the maximum EDOF of OAM multiplexing in radio and acoustic communications is equal to the azimuthal resolution of UCA.

**Figure 4:**
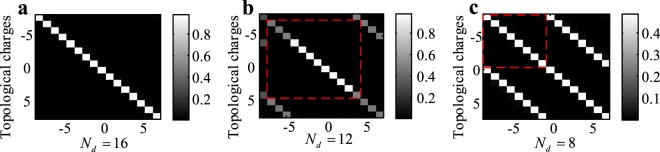
Use UCA_1×*Nd*_ to detect different OAM modes. The normalized spiral spectrum of the OAM modes detected (**a**) by UCA_(1×16)_; (**b**) by UCA_(1×12)_; (**c**) by UCA_(1×8)_.

Secondly, we analyze the physical resources of UCAs when they are used as OAM mode detectors. It was demonstrated that different OAM modes can be recognized by UCA which consists of uniform circular arranged sub-detectors^13,30^. The maximum distinguishable OAM modes of the UCA can be obtained by verifying the mutual orthogonality between detected OAM modes. Assume a single-ring ${{\rm{UCA}}}_{(1\times {N}_{d})}$ comprises of *N*_*d*_ detectors, and the *n*th detector is placed at (*r*, *ϕ*_*n*_) of the receiver plane. The OAM mode *l* detected by *n*th sub-detector is given by

4 $${u}_{d}(r,z,{\phi }_{n},l)={P}_{n}\exp (i\omega t-ikz+i{\phi }_{r})\exp (-il{\phi }_{n}),$$

where *P*_*n*_ is the amplitude, *z* is the transmission distance, *ϕ*_*r*_ contains all relevant phase lag, ${\phi }_{n}=\frac{2{\rm{\pi }}n}{{N}_{d}}$ is the azimuthal position of *n*th detector of receiver plane. Because of the rotational symmetry of OAM modes, ${P}_{1}={P}_{2}=\ldots ={P}_{{N}_{d}}$. The inner product between different detected OAM modes is given by

5 $$\begin{array}{ccc}{u}_{d}(r,z,m)\cdot {u}_{d}^{\ast }(r,z,k) & = & {\sum }_{n=1}^{{N}_{d}}{P}_{n}^{k}{P}_{n}^{m}exp[-i(m-k){\phi }_{n}]\\  & = & \{\begin{array}{cc}0 & m-k\ne \kappa {N}_{d}\\ {N}_{d}|{P}_{n}^{k}{|}^{2} & m-k=\kappa {N}_{d}\end{array}\end{array}$$

where *κ* is an unbounded integer. Eq.  (5) suggests that the maximum distinguishable OAM modes of the single-ring ${{\rm{UCA}}}_{(1\times {N}_{d})}$ is equal to the number of detectors (see methods). The examples of single-ring UCA(1 × *N*_*d*_) are shown in Fig. 4. The spiral spectrums of the OAM modes detected by single-ring UCA detectors verify that the maximum resolution of OAM modes equals to the number of elements. For the multi-ring UCA and unequally spaced UCA used as OAM mode detectors, we can determine their maximum distinguishable OAM modes by converting them into single-ring UCA, as we explained before. Thus, for UCA used as OAM mode detectors, their physical resources are equal to the azimuthal resolution of single-ring UCA as well.

Therefore, in OAM multiplexed radio or acoustic communications, if the UCAs are used as both OAM mode generator and detector, the maximum EDOF is equal to the minimum azimuthal resolution of the UCAs. Unlike conventional MIMO communications, which fully utilizes the physical resources, OAM multiplexing utilizes only the physical resources in the azimuth direction. Hence, from the aspect of maximum EDOF, OAM multiplexing can be seen as a weakened version of MIMO.

## EDOF decay and capacity limit induced by array element deviation

Although OAM modes can be generated by antenna arrays and realize multiple independent transmitted data streams as canonical MIMO communications do, it was controversial to consider OAM multiplexing as a subclass of canonical MIMO^30,31^. Especially from the perspective of antenna array arrangements, the most significant difference between OAM multiplexing and canonical MIMO is that OAM multiplexing requires the rotational symmetry of antenna array to ensure the helical phase pattern of signal and the mutual orthogonality between OAM modes, while canonical MIMO only requires the minimum distance between adjacent antennas. If there is a flaw in OAM mode generator, induced by array element deviation, the generated OAM modes will lose the mutual orthogonality and become impure. For the convenience of description, we use the IOAM modes to represent the impure OAM modes. It is recognized that IOAM modes are the superposition of numerical pure OAM modes including both lower order OAM modes and higher order OAM modes^17^. It is also known that, the power attenuation of lower order OAM modes is lower than those of higher OAM modes, and such power attenuation difference grows rapidly as the transmission distance increases^30^. That will cause the normalized spiral spectrum of IOAM modes to gradually concentrate in lower order OAM modes and make the transmitted IOAM modes inseparable. Given that, even a slight imperfection of UCA may eventually lead to a significant EDOF decay, the capacity of OAM multiplexing in radio and acoustic domain will be limited.

**Figure 5:**
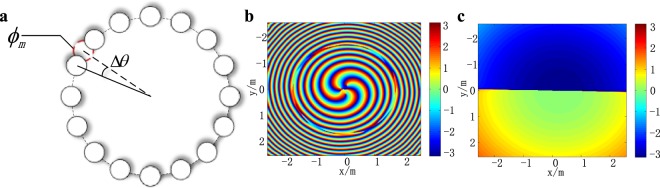
(**a**) Imperfectly arranged UCA_(1×16)_, where Δ*θ* is the azimuthal deviation and $\Delta \theta =\frac{2\pi }{160}$; (**b**) phase pattern of the IOAM mode *l* =−4 under transmission distance *z* = 20*λ*; (**c**) phase pattern of the IOAM mode *l* = −4 under transmission distance *z* = 2 × 10^3^*λ*.

To verify this, we analyzed the normalized spiral spectrum of IOAM modes generated by imperfectly arranged UCA_1×16_ under different transmission distances. Firstly, we assume that the *m*th element of UCA has a small azimuthal deviation Δ*θ*, as shown in Fig. 5(a). Δ*θ* would result in a flaw of the helical phase pattern of IOAM modes, as shown in Fig. 5(b). When the transmission distance *z* = 20*λ*, the phase flaw is inconspicuous, and the phase pattern of IOAM modes approximates to a ideal helical profile. However, as shown in Fig. 5(c), if the transmission distance is increased to *z* = 2 × 10^4^*λ*, the phase pattern is completely different from the original helical profile. And in fact, it approximates to the phase pattern of the superposition of ideal LG mode *l* = 1 and *l* = −1 (see supplementary). The IOAM mode *l* generated by UCA_(1×16)_ is given by

6 $${u}_{e}(r,z,\phi ,l)=\frac{{A}_{m}\lambda }{4\pi z{{\rm{{\prime} }}}_{m}}\exp (i\omega t-ikz{\rm{{\prime} }})\exp (-il{\phi }_{m})+{\sum }_{n=1,n\ne m}^{16}\frac{{A}_{n}\lambda }{4\pi z{{\rm{{\prime} }}}_{n}}\exp (i\omega t-ikz{\rm{{\prime} }})\exp (-il{\phi }_{n}),$$

**Figure 6:**
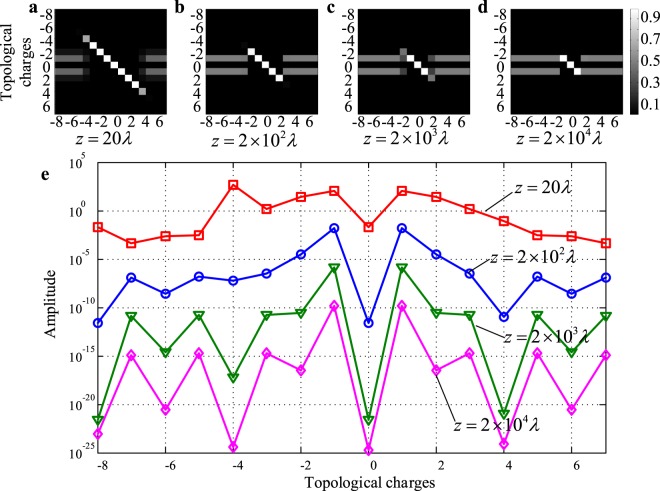
Normalized spiral spectrum of IOAM modes under transmission distance, (**a**) *z* = 20*λ*; (**b**) *z* = 2 × 10^2^*λ*; (**c**) *z* = 2 × 10^3^*λ*; (**d**) *z* = 2 × 10^4^*λ* respectively; (**e**) spiral spectrum of IOAM mode *l* = −4 under the transmission distance *z* = 20*λ*, *z* = 2 × 10^2^*λ*, *z* = 2 × 10^3^*λ* and *z* = 2 × 10^4^*λ*. The radius of UCA_(1×16)_ is *r* = 2*λ* and the radius of receiver aperture is *r*_*d*_ = 2*λ* and $\Delta \theta =\frac{\pi }{80}$.

The normalized spiral spectrum of *u*_*e*_(*r*, *z*, *ϕ*, *l*) under different transmission distances is shown in Fig. 6(a–d). One can see that the low order IOAM modes perform better in maintaining modes purity than high order IOAM modes do because of their lower power attenuation. If the transmission distance is further increased, the signal power would concentrates in OAM modes *l* = ±1. In addition, we discover that the increase of Δ*θ* will cause the decrease of EDOF as well (if the target OAM mode is still the main component of the detected signal, the emitted impure OAM mode is viewed as an EDOF). Table 1 lists the EDOF of the imperfectly arranged UCA_(1×16)_ under different azimuthal deviations Δ*θ*. It shows that for every ten times increase in Δ*θ*, the EDOF decreases by 2. Interestingly, the decaying process stops when EDOF decreases to 3, no matter how long the transmission distance and how large the Δ*θ* are. The corresponding three effective IOAM modes are *l* = 0 and *l* = ±1 respectively, as shown in Fig. 6(d).

**Table 1:** EDOFs of imperfectly arranged UCA_(1×16)_ under different transmission distance.

Δ*θ*(rad)/distance	*z* = 20*λ*	*z* = 2 × 10^2^*λ*	*z* = 2 × 10^3^*λ*	*z* = 2 × 10^4^*λ*
0	16	16	16	16
$0.01\times \frac{2\pi }{16}$	11	7	5	3
$0.1\times \frac{2\pi }{16}$	9	5	3	3
$1\times \frac{2\pi }{16}$	7	3	3	3

To figure out the decaying process of the EDOF, we analyzed the normalized spiral spectrum of IOAM mode *l* = −4 under different transmission distances, as shown in Fig. 6(e). When the transmission distance *z* = 20*λ*, the OAM mode *l* = −4 is the main component of IOAM mode *l* = −4, and the spiral spectrum has two lower peak value at *l* = ±1. Further increase the transmission distance, the power of OAM mode *l* = −4 will rapidly attenuate and the lower order OAM modes *l* = ±1 gradually become the primary components of the IOAM mode *l* = −4.

**Figure 7:**
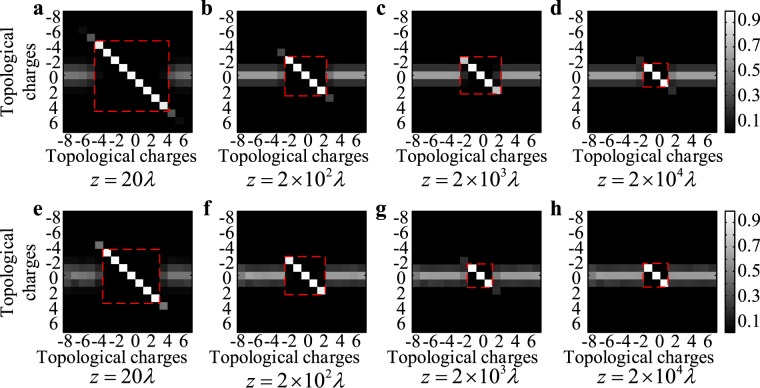
(**a**) to (**d**) depict the normalized spiral spectrum of IOAM modes under transmission distance *z* = 20*λ*, *z* = 2 × 10^2^*λ*, *z* = 2 × 10^3^*λ* and *z* = 2 × 10^4^*λ* respectively, when sole element of UCA_(1×16)_ has a random positional deviation; (**e**) to (**h**) depict the normalized spiral spectrum of IOAM modes under transmission distance *z* = 20*λ*, *z* = 2 × 10^2^*λ*, *z* = 2 × 10^3^*λ* and *z* = 2 × 10^4^*λ* respectively, when all elements of UCA_(1×16)_ have a random positional deviations. The random positional deviation *N*_*X*_ and *N*_*y*_ subject to normal distribution $N(0,{(1.133\times 1{0}^{-3})}^{2})$.

In addition, we find that even if the array element deviation comprises of the azimuthal deviation and the radial deviation, the above obtained results are still valid. For example, Fig. 7(a–d) shows that when the UCA has one element with a random positional deviation, the EDOF decays as the transmission distance increases. Moreover, the signal power would concentrate in OAM mode *l* = 0 instead of in OAM modes  ±1. As shown in Fig. 7(e–h), when all elements of the UCA have a random positional deviation, the EDOF decays faster. In both of these two conditions, OAM modes *l* = 0 and *l* = ±1 perform most robustly in maintaining the mode purity after long-distance transmission, and the EDOF decay stops when the EDOF decreases to 3.

The above examples show the EDOF decay induced by arrangement flaw of UCAs. The OAM modes generated by the imperfectly arranged UCA are inseparable at different transmission distances because the signal power of OAM modes consistently concentrate in OAM modes *l* = 0 and *l* = ±1. Although at near field, the element deviation is small enough to be negligible, the EDOF decays rapidly at far field as the transmission distance increases. Since the receiver cannot recognize the transmitted OAM modes correctly, this effect will severely impact the EDOF of generalized OAM multiplexing, such as OAM index modulation^32^ and OAM coding^13^.

Given the EDOF and information capacity are sensitive to the arrangement flaw and the transmission distance, the promising channel capacity and the far-field application of OAM multiplexing are challenged. To mitigate this effect, the lower-order OAM modes should be preferably applied as data carrier, because they have lower power attenuation and are better in maintaining mode purity. Moreover, since OAM multiplexing is seen as a subset of convention MIMO^30^, the element deviation induced EDOF decay might be mitigated by using the channel equalization methods as being used in MIMO system, such as least squares (LS) and minimum mean square error (MMSE).

## Conclusion

Thus far, we discuss the physical resources of UCAs and the EDOF of OAM multiplexing in radio and acoustic communications. We discover that the physical resources of UCAs, used both as OAM mode generator and detector, comprise of the the azimuthal resolution and radial resolution. The former limits the number of effective OAM modes and the later limits the radial intensity variation. Since the mutual orthogonality of OAM modes derives from the transverse helical phase pattern $\exp (-il\phi )$ which only depends on the azimuthal phase variation, the maximum of EDOF of OAM multiplexed communications is equal to the azimuthal resolution of UCA. If multi-ring UCAs are implemented as transceivers, the maximum EDOF of OAM multiplexed communications is less than conventional MIMO’s.

In practical OAM multiplexed communications, due to the inevitable flaws of real UCAs, the near-field negligible flaw will induce a significant EDOF decay in far field. More generally, even the UCA is perfectly arranged, the OAM modes will always be disturbed by ambient noises, such as turbulent medium, refraction and reflection, all of which cause the OAM modes imperfect. Therefore, the performance of far-field OAM multiplexing is unsecured. As far as we can perceive, one method to mitigate such effect is to use the lower order OAM modes as data carrier, but this will lead to the loss of its advantages in performance. Given that, the OAM multiplexing might be improper in far-field radio and acoustic communications.

## Methods

### The maximum of mutual orthogonal modes

The inner product between different OAM modes is given by Eq.  (3). To determine the maximum mutual orthogonal modes, we assume a set given by

7 $${U}_{s}=[{u}_{s1},{u}_{s2}\,\ldots \,{u}_{sk}\,\ldots \,{u}_{s{N}_{t}}],$$

where *u*_*sk*_ = *u*(*r*, *z*, *ϕ*, *s*_*k*_) is the OAM mode *l* = *s*_*k*_ which is generated by a single-ring UCA_1×*Nt*_, *s*_*k*_ = *s*_*k*−1_ + 1. Base on Eq.  (3), all elements belonging to *U*_*s*_ are mutually orthogonal. Suppose a *u*_*n*_ = *u*(*r*, *z*, *ϕ*, *n*) which subjects to *u*_*n*_ ∉ *U*_*s*_, *n* < *s*_1_ or $n > {s}_{{N}_{t}}$ and *u*_*n*_ is orthogonal to all elements, hence

8 $${u}_{n}\cdot {u}_{sk}^{* }=0,\forall {u}_{sk}\in {U}_{s},$$

For arbitrary integer *n*, it is easy to find a *s*_*n*_ satisfying *n* = *κN*_*t*_ + *s*_*n*_, where *κ* is an unbounded integer and *s*_1_ ≤ *s*_*n*_ ≤ *s*_*Nt*_. Since the inner product between *u*_*n*_ and *u*_*sn*_ is non-zero, this result contradicts the above assumptions. Hence, the maximum mutually orthogonal OAM modes generated by single-ring ${{\rm{UCA}}}_{(1\times {N}_{t})}$ is equal to *N*_*t*_.

### Demonstration of that the impure OAM modes are the superposition of two pure OAM modes

Assume that the UCA transmitter consists of *N*_*t*_ elements. On the transmitter plane, to generate the OAM mode $l=\frac{{N}_{t}}{2}$, the emitted signal is $A\exp (-i\omega t)\times {[\exp (-i\pi ),\exp (-i2\pi ),\ldots ,\exp (-i{N}_{t}\pi )]}^{{\rm{T}}}$. The spiral spectrum (here the spiral spectrum can be seen as the spatial version of conventional frequency spectrum) of this generated OAM modes is given by

9 $$\begin{array}{ccc}F(l) & = & A\exp (-i\omega t){\sum }_{n=1}^{{N}_{t}}\exp (-in\pi )\exp \left(-iln\frac{2\pi }{{N}_{t}}\right)\\  & = & \left\{\begin{array}{cc}0 & l\ne \kappa {N}_{t}+\frac{{N}_{t}}{2}\\ A{N}_{t}\exp (-i\omega t) & l=\kappa {N}_{t}+\frac{{N}_{t}}{2}\end{array}\right.\end{array}$$

where *κ* is an integer. The spiral spectrum suggests that this impure OAM mode $l=\frac{{N}_{t}}{2}$ is the superposition of pure OAM modes $l=\frac{{N}_{t}}{2}$ and $l=-\,\frac{{N}_{t}}{2}$. However, since the impure OAM mode $l=\frac{{N}_{t}}{2}$ is orthogonal to other pure OAM modes, this impure mode is efficient to carry an independent data stream as other EDOF do.

### Spiral spectrum of detected signal

The spiral spectrum can depict the composition of detected OAM modes. The spiral spectrum of OAM mode *u*(*r*, *z*, *l*) is obtained by calculating the inner product between *u*(*r*, *z*, *l*) and helical phase term $\exp (il\phi )$. The spiral spectrum value at OAM mode *l*_*d*_ is given by^24,33^

10 $$S({l}_{d})={\int }_{0}^{R}{\int }_{0}^{2\pi }r{\rm{d}}r{\rm{d}}\phi u(r,z,l)\times \exp {(-i{l}_{d}\phi )}^{\ast },$$

where *R* is the receiver aperture size.

### The distance between the arbitrary two points in cylindrical coordinate

**Figure 8:**
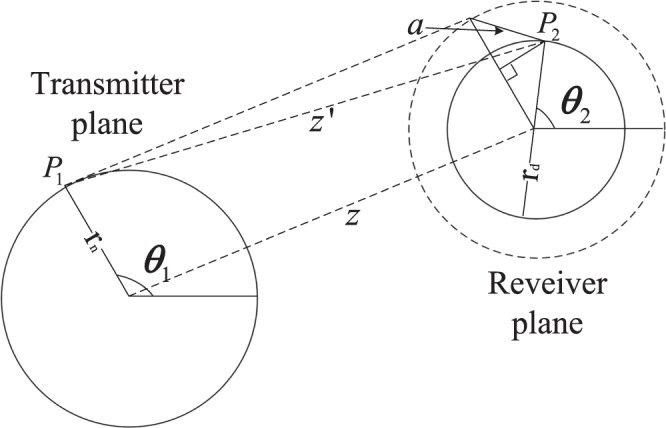
The distance between two points in cylindrical coordinate.

The distance is shown in Fig. 8. As shown in Fig. 8, *a*^2^ is given by

11 $$\begin{array}{ccc}{a}^{2} & = & {[{r}_{n}-{r}_{d}\cos ({\theta }_{1}-{\theta }_{2})]}^{2}+{[{r}_{d}\sin ({\theta }_{1}-{\theta }_{2})]}^{2}\\  & = & {r}_{n}^{2}+{r}_{d}^{2}-2{r}_{n}{r}_{d}cos({\theta }_{1}-{\theta }_{2}).\end{array}$$

Hence, the distance between two point is $z{\prime} =\sqrt{{z}^{2}+{r}_{n}^{2}+{r}_{d}^{2}-2{r}_{n}{r}_{d}cos({\theta }_{1}-{\theta }_{2})}$. Simplifying $z{\prime}$ yields $z{\prime} =z\sqrt{1+\frac{{a}^{2}}{{z}^{2}}}$. When the transmission distance *z* >> *a*, $\frac{{a}^{2}}{{z}^{2}}\approx 0$, expanding $z{\prime}$ to Maclaurin series yields $z{\prime} \approx z+\frac{{a}^{2}}{2z}$.

### Radial intensity variation of OAM modes generated by UCA

**Figure 9:**
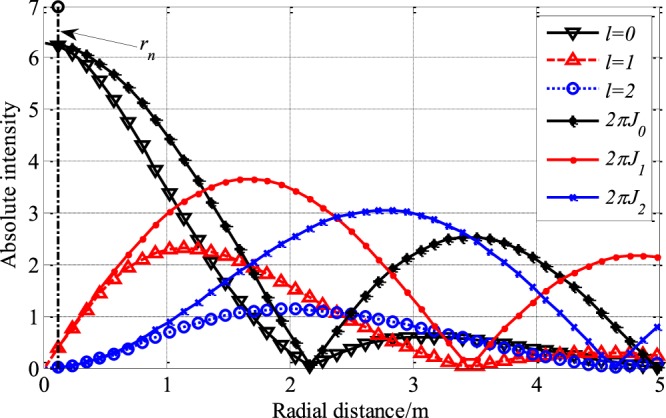
Radial intensity distribution, where *λ* = 0.1133 *m*, *z* = 100*λ*, *r*_*n*_ = *λ* and $\frac{A\lambda }{4\pi z}=1$. *l* = 0, 1, 2 denote the OAM modes generated by UCA_1×*∞*_ and *J*_0_, *J*_1_, *J*_2_ denote the absolute value of 0, 1, 2 order first kind Bessel function respectively.

In order to figure out why different power attenuations occur to the OAM modes, we analyzed the radial intensity variation of the OAM modes. Under the assumption that a single-ring ${{\rm{UCA}}}_{1\times {N}_{t}}$ with radius *r*_*n*_, the OAM modes generated by the ${{\rm{UCA}}}_{(1\times {N}_{t})}$ are given by Eq. (1). Assume the topological charge *l* subjects to *l* < *fracN*_*t*_2, the OAM modes generated by ${{\rm{UCA}}}_{(1\times {N}_{t})}$ should approximate to those generated by ${{\rm{UCA}}}_{(1\times \kappa {N}_{t})}$, where *κ* is a positive integer (the OAM mode generated by different UCAs see supplementary). If *N*_*t*_ → *∞*, the OAM modes generated by the UCA_(1×*∞*)_ is given by

12 $$u({r}_{d},z,{\theta }_{2},l)={\int }_{0}^{2\pi }\frac{A\lambda }{4\pi z{\prime} }\exp (-i\omega t-il{\theta }_{1}-ikz{\prime} )d{\theta }_{1},$$

where, *A* is the amplitude of the point of ${{\rm{UCA}}}_{1\times {N}_{t}}$. Substituting $\frac{A}{z{\prime} }\approx \frac{A}{z}$ and $z{\prime} \approx z+\frac{{a}^{2}}{2z}$ to Eq. (12) yields the following :

13 $$\begin{array}{ccl}u({r}_{d},z,{\theta }_{2},l) & = & \frac{A\lambda }{4\pi z}\exp (-i\omega t){\int }_{0}^{2\pi }\exp \left(-il{\theta }_{1}-ik\left(z+\frac{{r}_{n}^{2}+{r}_{d}^{2}-2{r}_{n}{r}_{d}cos({\theta }_{1}-{\theta }_{2})}{2z}\right)\right)d{\theta }_{1}\\  & = & \frac{A\lambda }{4\pi z}\exp (-i\omega t)\exp (-ikz)\exp \left(-ik\frac{1+{\gamma }^{2}}{2\zeta }\right){\int }_{0}^{2\pi }\exp \left(-i\left(l{\theta }_{1}-k\frac{\gamma cos({\theta }_{1}-{\theta }_{2})}{\zeta }\right)\right.d{\theta }_{1},\end{array}$$

where $\gamma =\frac{{r}_{n}}{{r}_{d}}$, $\zeta =\frac{z}{{r}_{d}^{2}}$. Since *l* is an integer, we can rewrite Eq.  (13) as

14 $$u({r}_{d},z,{\theta }_{2},l)=\frac{A\lambda }{4\pi z}\exp (-i\omega t)\exp (-ikz)\exp \left(-ik\frac{1+{\gamma }^{2}}{2\zeta }\right)\exp (-il{\theta }_{2})2\pi {J}_{l}\left(\frac{k\gamma }{\zeta }\right),$$

where ${J}_{l}\left(\frac{k\gamma }{\zeta }\right)$ is the first kind *l* order Bessel function, and $\exp (il{\theta }_{2})$ is the helical phase term. And, when *z* >> *a*, the accurate radial intensity variation function is given by Eq. (12) and the approximate representation is given by Eq. (14). As shown in Fig. 9, when *r*_*d*_ is small, the radial intensity variation is very close to 2*πJ*_*l*_. It can be seen that the power attenuations of different OAM modes are induced by divergence differences.

## Supplementary information

Supplementary information.
