# Supplementary material for: The Limits of Effective Degrees of Freedom in UCA based Orbital Angular Momentum Multiplexed Communications
Source: Sci Rep. 2020 Mar 23;10:5216. doi: 10.1038/s41598-020-61329-z (PMC7090009; doi:10.1038/s41598-020-61329-z)
Supplement: Supplementary file 1 — Supplementary information. [file 41598_2020_61329_MOESM1_ESM.pdf]

Supplementary information of ”The Limits of Effective Degrees of Freedom in UCA based Orbital Angular Momentum Multiplexed Communications”

Zhipeng Li[1], Fengzhong Qu[1,*], Yan Wei[1], Guowei Yang[2], Wen Xu[1], and Jing Xu[1]

[1]Ocean College, Zhejiang University, Zhoushan, Zhejiang 316021, China

[2]School of Communication Engineering, Hangzhou Dianzi University, Hangzhou, Zhejiang 310000, China

[*]jimqufz@zju.edu.cn

1 Intensity distribution and phase pattern of the superposition of Laguerre-Gaussian modes

a The intensity distribution and helical pattern of the superposition of LG modes $l = \pm 4$ are shown in Figure 1(a) and 1(b); those of the $\text{UCA}_{(1\times 8)}$ generated OAM modes $l = \pm 4$ (these two ”OAM modes” sharing same intensity distribution and phase pattern) are shown in figure 1(c) and 1(d). Both their intensity distribution comprise eight discrete sections, the difference is that the former intensity distribution is single-ring, while the later one is multi-ring. Their phase patterns also comprise eight discrete phase regions, the stagger regions correspond to the dark regions shown in the intensity distributions. The normalized spiral spectrum verifies the result that $\text{UCA}_{1\times 8}$ generated OAM modes $l = \pm 4$ can be decomposed into two ideal OAM mode $l = +4$ and $l = -4$. This stagger phase pattern was also observed in previous studies on mm-wave OAM[1].

b The intensity distribution and phase pattern of the superposition of LG modes $l = \pm 1$ are shown in figure 2(a) and 2(b). Figure 2(c) and 2(d) depict the intensity distribution and phase pattern of the OAM modes generated by imperfectly arranged UCA. Both their intensity distribution comprise two independent parts, and the phase pattern are segmented by a straight line. The intensity distribution and phase pattern shown in figure 2(c) and 2(d) approximate to the central part of the superposition of LG modes $l = \pm 1$.

2 Different UCAs generated OAM mode

As shown in figure 3, the OAM mode $l = -3$, generated by $\mathrm{UCA}_{1\times 16}$, $\mathrm{UCA}_{1\times 32}$ and $\mathrm{UCA}_{1\times 64}$ respectively, has a multi-ring intensity distribution and a helical phase pattern. The difference is that the intensity distribution shown in figure 3(a) is not a smooth ring profile, since the $\mathrm{UCA}_{(1\times 16)}$ has a lower azimuthal resolution. One can see that the different UCAs generated OAM modes are approximative, if the N_t is several times larger than the absolute value of topological charges of OAM modes.

1. Edfors, O. & Johansson, A. J. Is orbital angular momentum (oam) based radio communication an unexploited area? *IEEE Transactions on Antennas and Propagation* **60**, 1126–1131 (2012).

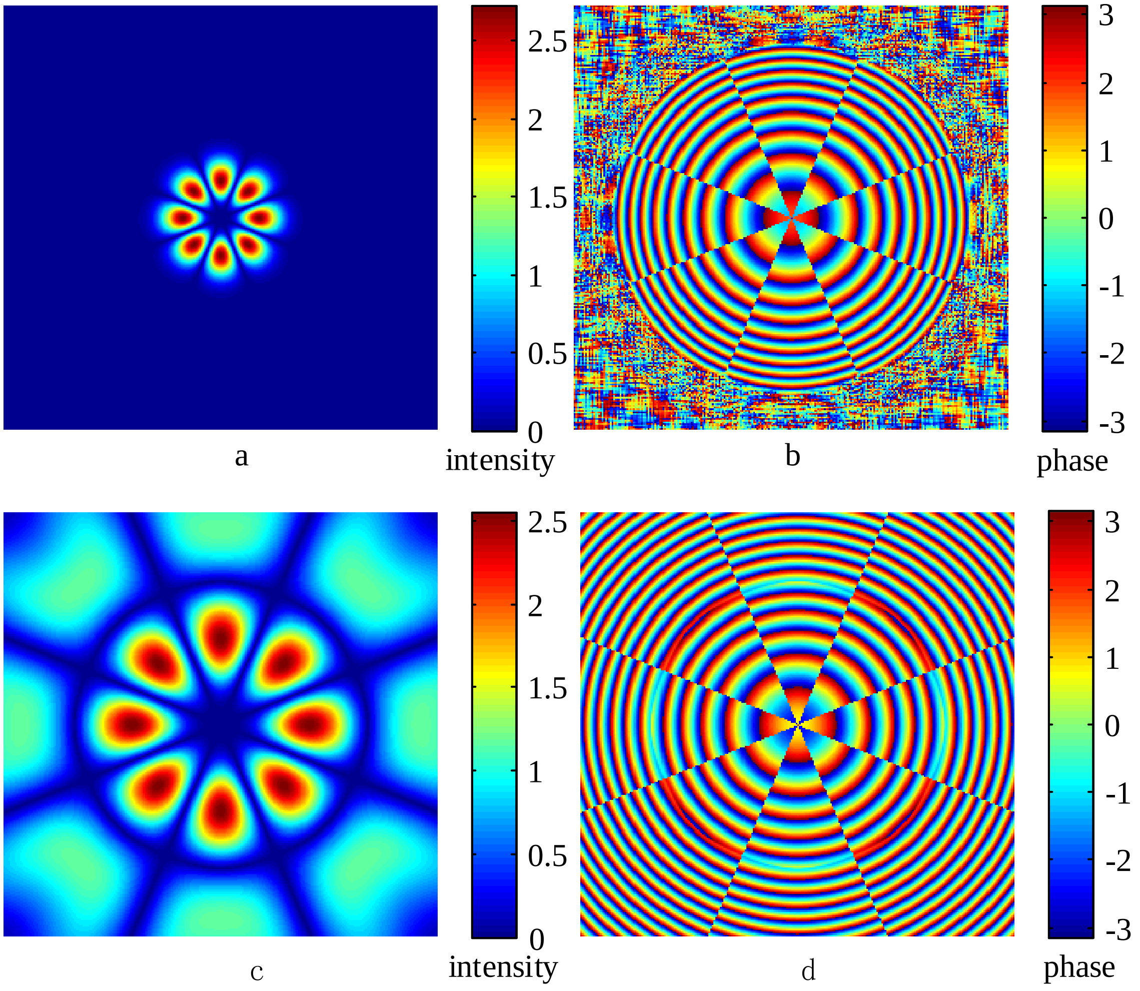

Figure 1: (a) and (b) depict the intensity distribution and the phase pattern of the superposition of LG modes $l = \pm 4$ respectively (the receiver plane is $1km$ away from the transmitter planar); (c) and (d) depict the intensity distribution and the phase patten of OAM modes $l = \pm 4$ which are generated by $\mathrm{UCA}_{1\times 8}$ respectively (the receiver plane is 20λ away from the transmitter plane).

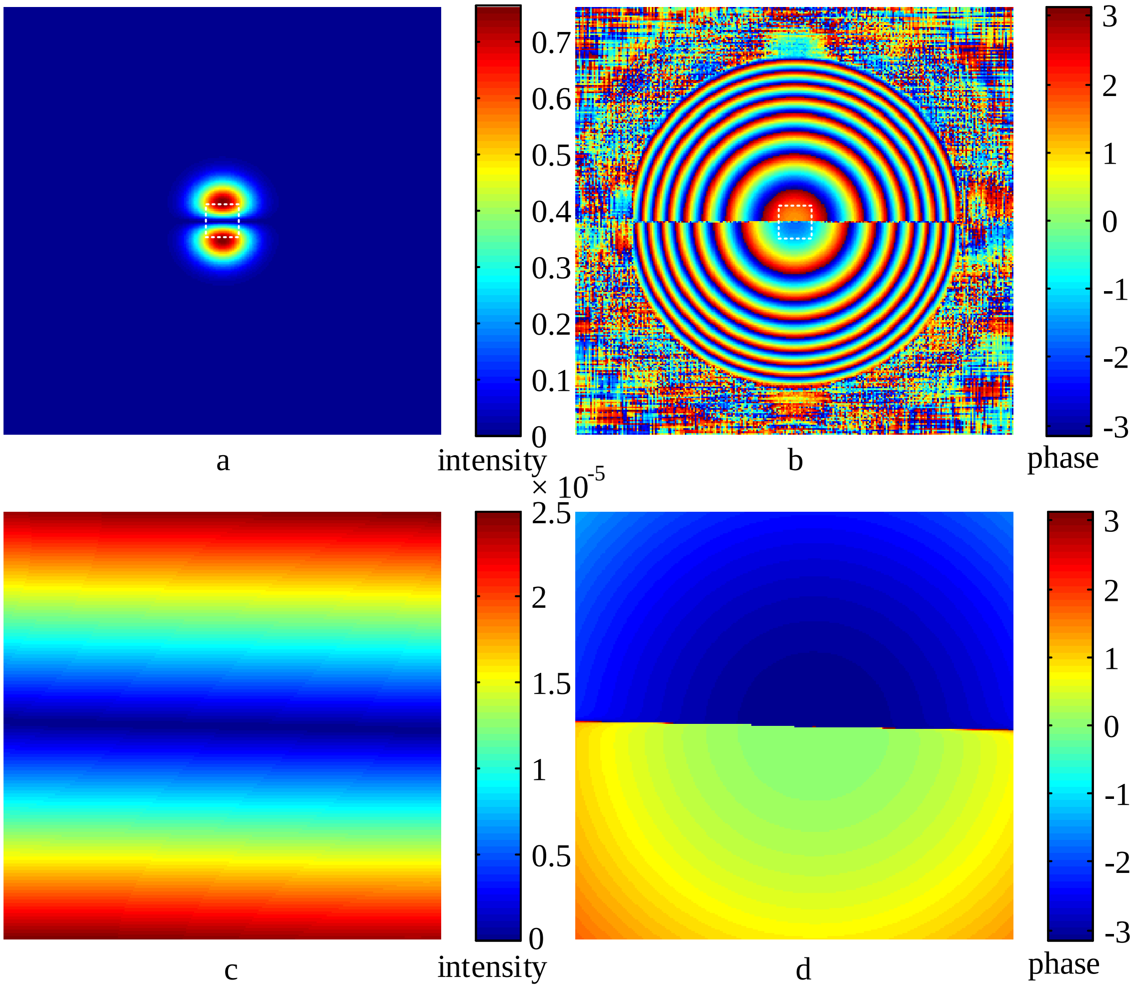

Figure 2: (a) and (b) depict the intensity distribution and phase pattern of the superposition of LG modes $l = \pm 1$(the receiver plane is $1km$ away from the transmitter planar); (c) and (d) depict the intensity distribution and phase pattern of the superposition of the OAM modes generated by the imperfectly arranged UCA(the receiver plane is $2 \times 10^3\lambda$ far away from the transmitter planar).

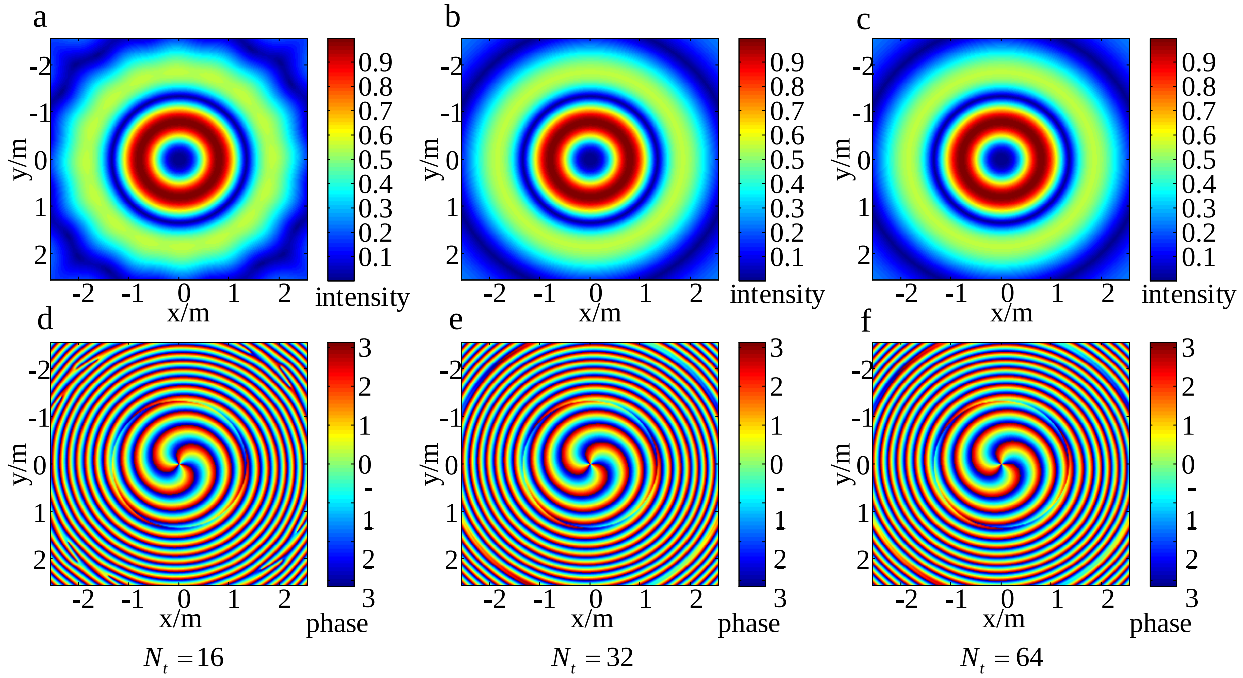

Figure 3: (a) to (c) depict the intensity distributions of the OAM mode $l = -3$ generated by $UCA_{(1\times16)}$,$UCA_{(1\times32)}$ and $UCA_{(1\times64)}$ respectively. (d) to (f) depict the corresponding helical phase patterns.
